# Supplementary material for: From sequence to enzyme mechanism using multi-label machine learning
Source: BMC Bioinformatics. 2014 May 19;15:150. doi: 10.1186/1471-2105-15-150 (PMC4229970; doi:10.1186/1471-2105-15-150)
Supplement: Additional file 2 — Java code of ml2db. Additional file ml2db_code.tar.gz contains the Java source code to run the multi-label machine learning experiments and save the results to database. The code’s Javadoc is included. [file 1471-2105-15-150-S2.zip › additional file 2/ml2db/utils/doc/uk/ac/ed/inf/utils/stringutils/StringUtils.html]

StringUtils


---


|  |  |  |
| --- | --- | --- |
| **Overview** \| **Package** \| **Class** \| **Use** \| **Tree** \| **Deprecated** \| **Index** \| **Help** | |  |
| **PREV CLASS**   NEXT CLASS | **FRAMES**    **NO FRAMES**     **All Classes** |  |
| SUMMARY: NESTED \| FIELD \| CONSTR \| METHOD | DETAIL: FIELD \| CONSTR \| METHOD |  |


---


## uk.ac.ed.inf.utils.stringutils Class StringUtils

```
java.lang.Object
  uk.ac.ed.inf.utils.stringutils.StringUtils
```

---

``` public class StringUtils extends java.lang.Object ```

Class

**Version:**
:   11 Mar 2008

**Author:**
:   Luna De Ferrari luna.deferrari-at-ed.ac.uk

---

**Constructor Summary**

`StringUtils()`


| **Method Summary** | |
| --- | --- |
| `static java.lang.String` | `booleanTo01String(java.lang.Boolean booleanValue)`             Returns "1" if Boolean.TRUE, "0" if Boolean.FALSE |
| `static boolean` | `containsPositiveInteger(java.lang.String string)`             Returns true if the string contains a parsable integer |
| `static java.lang.String` | `doubleQuote(java.lang.String text)`             Enclose text in double quotes |
| `static java.lang.String` | `doubleQuoteIfNeeded(java.lang.String text)`             Enclose text in double quotes if it contains non-alphanumerical characters such as . |
| `static java.lang.String` | `fromInputStreamToString(java.io.InputStream in)` |
| `static java.lang.String` | `getSerialNumericalEnding(java.lang.String baseString, java.lang.String currentString)`             Get a progressive +1 numerical ending. |
| `static boolean` | `isDash(java.lang.String string)`             check if the string is a dash |
| `static boolean` | `stringIsNullOrEmpty(java.lang.String string)` |

**Methods inherited from class java.lang.Object**

`equals, getClass, hashCode, notify, notifyAll, toString, wait, wait, wait`

**Constructor Detail**

### StringUtils

```
public StringUtils()
```


**Method Detail**

### getSerialNumericalEnding

```
public static java.lang.String getSerialNumericalEnding(java.lang.String baseString,
                                                        java.lang.String currentString)
```

:   Get a progressive +1 numerical ending. Eg. if base string is "n" and current string is "n12",
    the next string will be "n13". Beware, the current string must be null or contain the base string + an integer.

    :   **Parameters:**: `baseString` -: `currentString` - if null, the returned string is "0" **Returns:**: the next (+1) serial string (base string with previous numerical ending + 1)

---


### booleanTo01String

```
public static java.lang.String booleanTo01String(java.lang.Boolean booleanValue)
```

:   Returns "1" if Boolean.TRUE, "0" if Boolean.FALSE

    :   **Parameters:**: `booleanValue` - **Returns:**: "1" if true, "0" if false

---


### containsPositiveInteger

```
public static boolean containsPositiveInteger(java.lang.String string)
```

:   Returns true if the string contains a parsable integer

---


### fromInputStreamToString

```
public static java.lang.String fromInputStreamToString(java.io.InputStream in)
                                                throws java.io.IOException
```

:   **Throws:**: `java.io.IOException`

---


### isDash

```
public static boolean isDash(java.lang.String string)
```

:   check if the string is a dash

---


### stringIsNullOrEmpty

```
public static boolean stringIsNullOrEmpty(java.lang.String string)
```

:   **Parameters:**: `string` - **Returns:**: true if a string is null or empty

---


### doubleQuote

```
public static java.lang.String doubleQuote(java.lang.String text)
```

:   Enclose text in double quotes

    :   **Parameters:**: `text` - **Returns:**: "text"

---


### doubleQuoteIfNeeded

```
public static java.lang.String doubleQuoteIfNeeded(java.lang.String text)
```

:   Enclose text in double quotes if it contains non-alphanumerical characters
    such as . or , or -

    :   **Parameters:**: `text` - **Returns:**: "text" if the text contains non alpha-numerical characters, text otherwise


---


|  |  |  |
| --- | --- | --- |
| **Overview** \| **Package** \| **Class** \| **Use** \| **Tree** \| **Deprecated** \| **Index** \| **Help** | |  |
| **PREV CLASS**   NEXT CLASS | **FRAMES**    **NO FRAMES**     **All Classes** |  |
| SUMMARY: NESTED \| FIELD \| CONSTR \| METHOD | DETAIL: FIELD \| CONSTR \| METHOD |  |


---
